# Components of Active Music Interventions in Therapeutic Settings—Present and Future Applications

**DOI:** 10.3390/brainsci12050622

**Published:** 2022-05-10

**Authors:** Lydia Schneider, Louisa Gossé, Max Montgomery, Moritz Wehmeier, Arno Villringer, Thomas Hans Fritz

**Affiliations:** 1 Max Planck Institute for Human Cognitive and Brain Sciences, Stephanstrasse 1A, 04103 Leipzig, Germany; montgomery@cbs.mpg.de (M.M.); wehmeier@cbs.mpg.de (M.W.); villringer@cbs.mpg.de (A.V.); 2 Centre for Brain and Cognitive Development, Birkbeck University of London, Malet Street, London WC1E 7HX, UK; louisa.gossee@gmail.com; 3 Institute for Psychoacoustics and Electronic Music (IPEM), Ghent University, Blandijnberg 2, 9000 Ghent, Belgium

**Keywords:** active music therapy, musical agency, physical exercise, rehabilitation

## Abstract

Musical interventions in therapy have become increasingly relevant for rehabilitation in many clinics. What was long known for physiotherapy training—that the agency of the participant is crucial and moving is much more efficient for rehabilitation success than being moved—has over recent years also been shown to be true for music therapy. Accumulating evidence suggests that active musical interventions are especially efficient at helping rehabilitation success. Here, we review various approaches to active music therapy. Furthermore, we present several components that allow for manipulating musical expressiveness and physical engagement during active musical interventions, applying a technology-based music feedback paradigm. This paper will allow for a transfer of insights to other domains of music-based therapeutic interventions.

## 1. Introduction to Active Music-Based Interventions

Active music therapy approaches have been used either on their own or as part of polytherapy (use of a treatment that combines two or more therapies) to treat a single condition or alleviate symptoms of several psychiatric or neurological disorders regarding cognitive, emotional, social, and motor functioning. We define active music therapy as a combination of music and therapy [1,2] where patients actively participate in making music rather than being passively exposed to music. The effects of active music interventions have been studied most extensively in the context of neuro-rehabilitation. Active music therapy can comprise several musical activities, including rhythmical training, musical instruments, and singing. Below, we review various approaches to active music therapy. Furthermore, we present several components that (from theoretical considerations outside the scope of the current manuscript) allow for manipulating musical expressiveness and physical engagement during active musical interventions. Importantly, while we report several studies in different categories of one-time interventions and training, we emphasize here that musical interventions, even if often very motivating and engaging to participants, are no miracle cure. Indeed, various studies have reported issues in making musical interventions work via a far transfer effect to create cognitive or physiological benefits for users [3]. This makes it especially important to gain further insight into which components of musical interventions are key to therapeutic gain. Furthermore, it is clear that musical interventions are not always equally beneficial in a clinical setting. For example, a downside of a musical intervention may be that increasing cognitive load as an effect of the music intervention may interfere with certain aspects of walking performance and increase the likelihood of freezing in patients with Parkinson’s disease [4].

### 1.1. Rhythmical Training

Neurologic music therapy (NMT) is a promising approach for supporting neurorehabilitation and facilitating functional recovery (see review by Altenmüller & Schlaug [5]). As an example, Thaut et al. demonstrated that a 30 min musical intervention session using a musical entrainment paradigm significantly improved mental flexibility and self-efficacy in patients with traumatic brain injury (TBI) [6]. A key component of the paradigm included exercises on rhythmic synchronization. However, patients did not improve in terms of memory or attention in this study. The effect size for this study was moderate at 0.3. Another type of music therapy utilizing rhythmic synchronization is rhythmic auditory stimulation (RAS). It requires synchronizing movements (e.g., footsteps) with a sound that can be “metronome-like” or complex music [7]. RAS was shown to be effective at increasing motor function in several impairments. Hemiparetic stroke patients showed improvements in gait and upper body rehabilitation following RAS [7]. In summary, using rhythmical training was shown to improve motor function in particular, though cognitive effects were reported occasionally.

### 1.2. Practice of Musical Instruments (Musical Training)

In a study using common musical instruments, auditory feedback through playing a keyboard was seen to increase leftward exploration in patients with spatial neglect [8]. Eleven patients with leftward spatial neglect were instructed to press all the white keys from the furthest on the right to the end of the keyboard. When the keys were coupled with the standard right to left scale, patients showed significant leftward exploration compared to silent or random unmatched sound conditions [8]. Another study used musical instruments in a 30 min intervention program four times per week with two chronic visual–spatial neglect patients. After the intervention program, the spatial awareness of the two patients had increased, and an improvement in tests assessing neglect at a one-week follow-up demonstrated a longer-lasting effect. Similarly, an eight-week piano tuition program for patients with mild traumatic brain injury and two healthy control groups was conducted, one of which joined the same program and the other did not [9]. The music training groups received two 30 min lessons a week and were instructed to practice at least 15 min a day. After eight weeks, both music-training groups showed a significant improvement in learning strategies, memorization, and retrieval of information as measured by the California Verbal Learning Task. Meanwhile, the healthy control training group also showed a significant improvement in the Stroop word/color test, but patients did not show an improvement on this task (reading speed, attention). The effect size for this study was 0.4. Another example where instrument training was shown to have a positive effect on motor skill recovery is in patients with a loss of control in hand and arm movements after a stroke. Those patients who were taught how to play an electronic drum set and piano, in comparison to a control group who received conventional treatment, showed improved motor control after training [10], as supported by a large effect size (0.89). Beyond improving motor function, actively making music either using voice or instruments was also reported to improve emotional functioning and perceived quality of life in patients with Parkinson’s disease (PD) who suffer from severe motor problems, such as bradykinesia [11] compared to a similar patient group engaging only in physical activity [2]. A meta-analysis that considered six RCTs also supported the notion that music therapy promoting active musical engagement (for activities that do not require a far transfer, i.e., that included elements of gait) would to a certain degree improve motor symptoms during walking in PD patients [12]. Results from several studies suggested that an improvement in several motor symptoms through music therapy was more prominent when the music had isochronous properties [13,14,15].

In summary, playing a musical instrument as part of the therapeutic process showed improvements in motor function across a range of neurological conditions. In addition, several studies also reported improvements in cognitive and socio-emotional domains, though these findings showed less cross-study correspondence than the motor findings.

### 1.3. Singing and Singing Combined with Rhythmical Exercise

Melodic intonation therapy, where after a stroke, patients are taught to sing simple melodic contours whilst tapping was shown to be effective at improving speech in non-fluent aphasics [16]. In people with dementia, musical leisure activities, including singing and listening to music, were reported to improve mood, orientation, remote episodic memory, and, to a lesser extent, attention and executive function. Singing also enhanced short-term and working memory and caregiver well-being, whereas listening to music had a positive effect on their quality of life [17]. In conclusion, regular musical leisure activities demonstrate several benefits in mild/moderate dementia and it might be advisable to use these in dementia care and rehabilitation. Furthermore, singing whilst walking was shown to improve PD patients’ gait [18]. A recent study [19] compared external (music listening) versus internal cueing (singing) in people with PD in a different study. They found that internal cueing improved gait velocity, cadence, and stride length when walking backward, and reduced the variability in backward and forward walking [19]. In comparison, external cueing reduced gait stability and improved other gait parameters minimally. However, some recent studies on different conditions showed more modest results. Peteresen and colleagues [20] reported an effect of music therapy in patients with recent cochlear implants, mostly on music-specific skills, such as music perception, but transfer skills in speech perception were also evident in the control group. Studies such as these, in combination with publication bias, need to be considered in any discussion of the merits of music therapy. Singing has also been shown to provide improvements in symptomatology in children with neurodevelopmental disorders. A recent study by Sharda and collegues [21] evaluated neurobehavioral outcomes of a musical intervention (improvisational approach through song and rhythm) in children with autism spectrum disorder (ASD). Fifty-one children (6–12 years) with ASD were randomly assigned to a musical intervention or non-musical intervention (behavioral intervention) group for 8–12 weeks. Social communication improved significantly in the musical intervention group as compared to the behavioral (and non-musical) intervention group. Furthermore, several physiological changes were observed. For example, resting-state functional connectivity between auditory and subcortical regions and fronto-motor regions was greater in individuals who received the musical intervention. Interestingly, the post-intervention brain connectivity was related to increased communication skills in the music group. In addition, connectivity between auditory and visual regions (a region known to be over-connected in ASD) was significantly lower in the musical intervention group compared to the non-musical intervention group. These results suggest that musical interventions have the potential to improve social communication skills, perhaps corresponding to changes in functional connectivity in children with ASD. Furthermore, the ability of children with ASD to recognize affective signals conveyed by music is a great resource and was argued to be enhanced by regular music therapy [22]. A recent Cochrane systematic review confirmed these effects of music therapy on individuals with ASD [23] which included ten randomized controlled trials and controlled clinical trials (165 participants in total).

In summary, singing similarly to instrument playing can lead to wide-ranging improvements in symptoms of neurological and neurodevelopmental disorders. Moreover, the combination with rhythmical movement seems to yield additional benefits in some patient groups.

### 1.4. Which Components of Active Musical Interventions Drive the Therapeutic Effects?

As outlined above, active music-based interventions range from simple rhythmical training (e.g., RAS) to complex musical training (e.g., practicing musical instruments) and are often combined with certain activities (e.g., walking, speaking) to increase the immediate positive effects on those activities. Active music-based interventions were shown to be applied in a wide range of psychiatric and neurological conditions, such as stroke, TBI, PD, or ASD. Beneficial effects of such interventions include improved cognitive, motor, and/or socio-emotional functioning in patients. These studies show the wide range of applicability of different types of active music-based interventions; however, it complicates identifying which components of active music-based interventions are responsible for the improvements observed in such diverse patient groups. Disentangling the underlying mechanisms is difficult, as active music intervention protocols differ greatly from each other. Furthermore, individual music preferences and skills dictate the choice of therapeutic music interventions and, therefore, may differ from patient to patient. Moreover, it may be therapeutically convenient to combine music with certain tasks, e.g., walking in PD patients, to facilitate those challenging tasks for patients. The term “active” music intervention therefore includes a range of different components that are relevant for driving certain therapeutic effects. These components may include the following: intensity of motor engagement (tapping, walking, dancing), level of physical exertion (depending on impairments of motor functioning, certain movements are more exhaustive for one patient than for another), level of emotional arousal and engagement, level of musical aptitude, level of creativity, level of agency/self-efficacy (improvising vs. playing musical pieces as required), level of social interaction (alone vs. dyadic vs. group interventions), and all possible interactions of these components. Separating the effects of those components from one another is challenging, in particular when it comes to physical activity. Physical activity during music therapy, and probably every process in making music, plays an important role. This is especially relevant when considering the distinction between music therapy that requires more or less active participation and exertion. Evidence demonstrates that physical exertion, as part of physical exercise, is a highly effective method to increase brain plasticity (e.g., Fernandes et al., 2017) [24]. Exercise is therefore regarded as a key intervention in rehabilitation [25]. Making music involves a significant amount of effort with respect to both exerting motor control and physical exertion while playing musical instruments. Thus, given that effort/exertion is an integral component of music-making, we argue that it is crucial to look at the effects of this parameter on neuroplasticity to better understand how musical activities will affect brain plasticity and behavioral modification. Furthermore, when designing music experiments that involve a certain degree of physical engagement, it is of utmost importance to ensure an experimental design that is well balanced with respect to physical engagement. For this purpose, a technology-based music-feedback intervention (Jymmin^®^, Leipzig, Germany) was developed, which allows for identifying and varying components of active musical interventions while precisely considering the amount of physical engagement (among other parameters, see below).

## 2. Technology-Based Music-Feedback Paradigm (Jymmin^®^)

A key point that was argued to differentiate a sound musical intervention paradigm from a less useful paradigm is to include appropriate control conditions or groups that engage in an activity to a similar degree to which they engage in the active musical intervention. A meta-analysis about the transfer effects of musical training on working memory identified that it is essential to rule out placebo effects, which is impossible if an active intervention is compared to a passive task that does not create positive expectations about training or excitement induced by a novel activity [26]. For example, the authors of this meta-analysis [26] performed a comparison analysis of effect sizes of musical intervention studies that either utilized an active control group or a passive group. Strikingly, they present a figure that seems to demonstrate that when control groups performed a similarly active task, then the effect sizes deteriorated dramatically (passive control group: effect size of 0.25 SD vs. active control group: effect size of 0.18 SD). The insights of Sala & Gobet [26] suggest a paradigm where the musical intervention should be compared to a control condition of equal activity levels wherever possible. Here, we want to introduce a paradigm with a focus on balancing the activity level and musical stimuli between experimental and control groups or conditions.

Naturalistic control settings with activities such as chess, drama classes, and learning a language can provide a useful contrast to most music-based interventions, as they are often an ecologically valid equivalent to the intervention. However, we recognize that machine-based control conditions that match the musical intervention as closely as possible in terms of activity levels and the content of the musical stimuli can allow for stronger conclusions in psychological research. Music-feedback exercise (MFE) operates on the principle of sonification, where movement is transformed into an auditory output, such that participants can musically and spontaneously control the output. Compared to a simple biofeedback paradigm with audio feedback (e.g., pitch sweep) to monitor the smoothness/tempo of a movement, MFE would more strongly emotionally involve participants. MFE achieves this by providing participants with a sense of musical agency and the ability to actively control music according to their personal aesthetic preferences.

**Figure 1:**
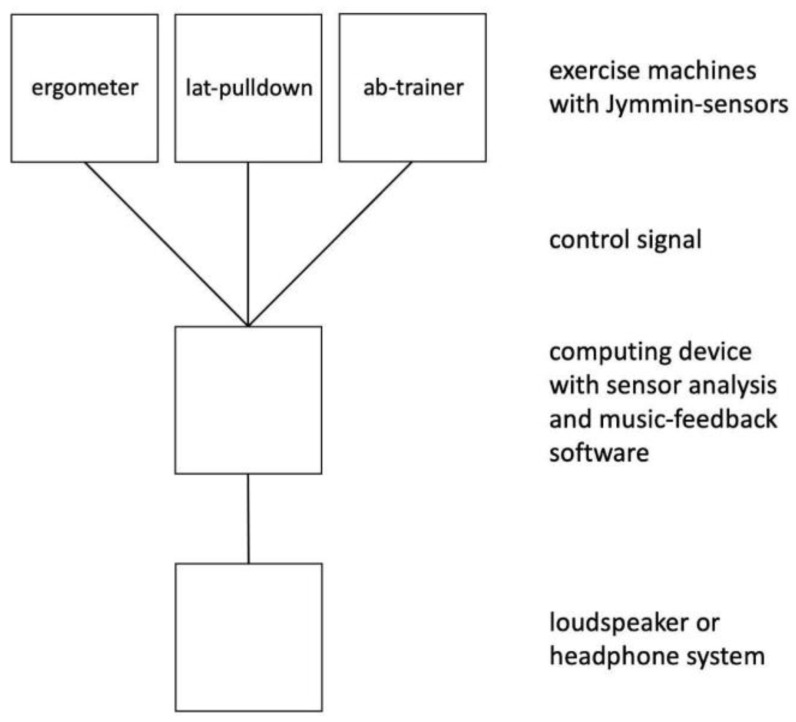
Jymmin setup.

In MFE, the aspect of physical exertion was shown to positively interact with the act of music-making, adding to the immersive experience during interventions (see below). For this, a sensor is often attached to a piece of exercise equipment. In those patients who already perceive simple body movements as strenuous, or when gymnastic movements are exercised, the body movements themselves may be monitored and mapped to music feedback. The sensor reports movement to a computer, tablet, or smartphone system that translates the sensor output into a music signal (see Figure 1 for a visual description). An MFE can either change continuously, e.g., by varying a cutoff filter, or can use thresholds (this means that participants must reach a certain threshold of force or work before the music signal changes) to translate the movement data into an auditory signal, or both. The setup previously used in MFE research was developed by Jymmin GmbH, which is a company specializing in the development and production of MFE solutions. A typical solution consists of three components, namely, (1) a sensor, (2) a movement analysis and music-processing device, and (3) a speaker. Below we provide details on how the components work.

A sensor can either be an onboard sensor system in available rehabilitation/sports exercise equipment or a wearable device. In the former category, a system with implemented music feedback is commercially available from medica THERA-Trainer. In the wearables category, Movesense™ sensors and sometimes smart devices and specifically developed sensors have been used. As computing devices, PC, MacOS, Android, or iOS have been used. The devices run with proprietary software developed by Jymmin GmbH that allows for the analysis of sensor signals and controls the musical output. The musical output is created from a set of pre-made musical “loops”, which can be several minutes long. Using patented software, the performer can navigate between such musical loops in accordance with their physical engagement, for example, blending in new loops with increased energy expenditure or multi-crossfading between several loops along a dimension of exertion. Using a composition tool developed by the Jymmin GmbH, this dimension of exertion can be coupled to one (single player) or several (multi-player) music “instruments” that can be played quite intuitively, also ranging from more relaxed versions of a musical loop to more energetic versions of the same or similar loop. This control of the musical output is thus achieved through a combination of setting thresholds for when the musical “loop” changed from one to another, and thresholds determining the onset and offset of an acoustic filter, which gradually changes the music.

The third and final stage of the MFE process is the processing of the musical output from a digital signal to an actual sound via speakers or headphones. In previous research, speaker systems with a good dynamic range and a subwoofer were used (e.g., Bose “S1 Pro System”) or medium-cost headphones (e.g., Urbanears Plattan). It is unknown what a minimum requirement for a sound system would be and if previously observed effects can be achieved if less sophisticated speakers, such as those found integrated into a smartphone, were to be used.

The music-feedback paradigm described above allows for investigating components of active musical interventions in a systematic manner because they either influence arousal during an intervention or how musically expressive participants perceive themselves to be. This also allows for controlling several factors that may have an influence on the outcome variables investigated by therapeutic studies. These methodological characteristics include the following: (1) Measurability of physical activity/engagement and the possibility to provide the same physical activity levels for the experimental and control group. (2) The physical engagement can be varied and adjusted in terms of individual physical effort in relation to individual physical capabilities. For this purpose, an individual range of movements can be calibrated to ensure a similar range of musical agency experience for all participants. (3) What is heard in the active music-making condition and passive music listening conditions can be balanced or controlled for very precisely (e.g., music created by active groups or in active conditions can be used as musical material in passive music listening groups or conditions). (4) The degree of control over the music can be precisely defined, for example, the number of musical instruments included. (5) The social experience during music-making can be systematically varied—e.g., through the number of interactive agents to create the music—if one plays alone, for example, or in a group with others like in a band. (6) An experience of musical agency (being in control of the musical output) can be achieved even for non-musicians and people naive to handling any type of musical instrument. (7) The musical material manipulated can be systematically varied, e.g., according to aesthetical preference, personal choice, or correspondence with the current personal emotional state. (8) The actuator (control signal) can be precisely chosen or varied, e.g., which muscles are used to make music.

## 3. Non-Clinical Studies Using Jymmin as an Active Music Intervention

In several non-clinical studies, we researched the effects of the previously described active music intervention (Jymmin) compared to passive music intervention that involves the same amount of physical engagement. We were therefore able to separate the effects of active, physically engaging music-making from being comparably physically active while listening to music in a systematic manner. This direct comparison of active and passive music intervention allowed for investigating the possible effects on cognitive, emotional, and physiological outcome measures while keeping the amount of physical engagement and exposure to musical stimuli controlled during the experiment.

### 3.1. Studies Addressing Components of Active Music Intervention on Motor Functioning/Subjectively Perceived Effort

In a study with sixty-three healthy participants (21 female, 42 male), the relationship between musical agency and perceived bodily effort was investigated [27]. Participants with professional experience in sport or fitness training were excluded, as well as professional musicians. Participants used fitness machines in groups of three. In the musical agency condition, the participants had the possibility of controlling instruments and other aspects of the music (rhythm, timbre) by moving (working out) on the fitness machines. The three machines were connected to a music production/composition setup that enabled the participants to make music interactively, similar to playing in a band. In the passive music condition, the same musical pieces were listened to while working out on the same fitness machines, but participants could not control the music themselves. Physiological and behavioral measures were assessed in order to investigate the effects of active music-making. Effects showed that while the amount of work performed on the fitness machines did not differ significantly between the two conditions, participants showed greater muscle efficiency during the music-making condition (higher level of metabolism in terms of oxygen consumption (WAER) in the condition without musical agency, as assessed by using spirometry). Additionally, the participants’ perceived exertion was significantly lower in the condition with musical agency.

In a follow-up study, it was investigated how the experience to be creative during the music-making condition influenced the perceived physical effort (Fritz et al., in preparation). Twenty-eight healthy participants (18 male, 10 female) performed a physical workout in combination with different musical interventions. None of the participants were professional athletes or musicians. All participants performed three different experimental conditions alone in a randomized order. In the first condition called “monotonous without beat”, participants performed stereotypical movement patterns on a lat-pulldown fitness machine, which were accompanied by simple acoustic feedback, namely, a pitch sweep, where the pitch corresponded to the extent participants pulled the weights; the further they pulled, the higher the pitch. The movements to be performed were simple pull movements with one onset and one offset of the movement (no holding/isometric movements for example or spontaneous change of direction in the course of the movement). In the second condition called “monotonous with beat”, participants performed similar movements on the same fitness machine with the same pitch sweep biofeedback, but they additionally listened to a drum beat that was not modified by the participants’ movements and was continuously playing during the condition. In a third condition called “creative with beat”, participants again performed similar movements on the same fitness machine with the same type of acoustic feedback (pitch sweep) and the beat playing. In addition, in the third condition, participants were also instructed to initiate the movements such that the simple acoustic feedback aesthetically fit best with the ongoing beat. In this way, participants were encouraged to be creative in a very controlled fashion and at a minimal level. “Exertion” scores following the condition “creative with beat” were significantly lower than those for the condition “monotonous without beat” and condition “monotonous with beat”, while there was no significant difference between the “monotonous without beat” and “monotonous with beat” conditions. Note, that the “creative with beat” condition had not been perceived to be more interesting, positive, or pleasant by the participants, which was also assessed during the experiment.

### 3.2. Studies Addressing Influences of Active Music Intervention on Emotional Parameters

Effects of an active music intervention as compared to passive music listening while keeping the amount of physical activity constant were compared in terms of mood changes (general mood and anxiety measures). Fifty-two participants (25 female, 27 male) took part in the experiment, where again, none of the participants were professional athletes or musicians [27]. Participants used fitness machines in groups of three. Two experimental conditions were compared using a cross-over design: in the musical agency condition, the participants had the possibility to control aspects of the music expressively by moving (working out) on the fitness machines so that their musical output combined to produce a music piece that was composed to fit together in tonality, rhythm, and timbre. In the passive music control condition, the same musical pieces (recordings of musical agency condition) were listened to while working out on the same fitness machines, but participants could not control the music with their movements. Results of this study showed that the musical agency condition increased mood (subscale of the Multidimensional Mood Questionnaire (MDMQ)) significantly compared to the passive music condition, with a moderate effect size of 0.2, as assessed using Pillai’s trace. Other subscales of the MDMQ did not differ (calmness vs. agitation, alertness vs. tiredness). Furthermore, an interaction effect of order and condition on the “calmness vs. agitation“ scale was observed such that when the musical agency condition was performed first, the level on this sub-scale increased significantly as compared to having the passive music condition performed first (possibly a hormonal effect lasting the duration of both conditions). No effects were observed in terms of changes in anxiety levels in this group of participants with low anxiety levels. In a follow-up study on the influence of an active music intervention on mood, underlying physiological mechanisms were indirectly investigated. We investigated whether the musical agency condition more strongly stimulated the release of endogenous opioids by applying a pain threshold paradigm [28]. The experimental setup was kept as similar as possible to the study on mood described above, except that participants used fitness machines in groups of two. Twenty-two participants (12 female, 10 male) took part in the experiment and performed both experimental conditions (see description above) in a cross-over design. As an outcome measure, pain tolerance was assessed by applying cold pressor tasks after each condition. Furthermore, general pain sensitivity and movement data were assessed in order to control for possible covariates. The effect size was η^2^ = 0.25. The results showed that pain tolerance was significantly increased following the musical agency condition as compared to the passive music condition. Participants tolerated the cold pain stimulation on average for five seconds longer after having performed the musical agency condition. A repeated measures ANCOVA was used in order to control for individuals’ general pain sensitivity, gender, and order of conditions. Furthermore, movement data (weight shift distance) showed that for one fitness machine (lat pulldown), the weight shift distance was significantly higher during the passive music condition. This is important to consider, as physical activity was previously shown to be positively correlated with pain tolerance [29,30,31,32]. These results about pain tolerance suggest a role for endogenous opioids in the mediating effects of music interventions. However, non-opioidergic mechanisms of analgesia, as well as psychological factors (e.g., attention, emotional state), may have also mediated the observed effect of an active music intervention on pain tolerance, factors which were not examined in the scope of the study.

### 3.3. Studies Addressing the Influence of Active Music Intervention on Cognitive Parameters

In a recent study, we also investigated the effects of active music interventions on cognitive capabilities [33]. Here, two active music interventions were introduced. One was the technology-based music-feedback intervention “Jymmin”, and another active music intervention consisted of a knob setup to make exactly the same music while turning knob controllers but without the strenuous physical engagement. The third experimental condition was listening to music while being physically active in a manner comparable to the Jymmin condition at the lat-pulldown machine. Therefore, this experimental setup allowed us to compare passive vs. active music interventions, as well as active music interventions with varying degrees of physical engagement. Note that the audible musical stimulation was similar in all three experimental conditions. Seventy-nine participants (44 female, 35 male) were randomly assigned to one of the experimental conditions and performed the music intervention in groups of three. Before and after having performed the intervention, participants were asked to complete a cognitive task known as the Guilford’s Alternative Uses Task [34], which measures divergent thinking capabilities. In this task, participants were asked to name as many uses as possible for either a bottle or a brick and had two minutes to complete the task. Results showed that divergent thinking was significantly increased after performing the music-feedback intervention with high physical engagement compared to the music intervention using a knob setup and the music listening condition. No significant difference was found between the other two conditions. This seems to indicate that a combination of both components of active music intervention, namely, musical agency and physical engagement, are necessary in order to increase divergent thinking capabilities. We think this finding could be relevant when investigating compensatory strategies after decline/loss in cognitive functioning and that in this scenario, creativity could play a key role. In combination with active music therapy, this boost in divergent thinking/creativity might account for some of the prior research findings that show the generalization effects of active music therapy.

## 4. Randomized Clinical Studies

### 4.1. Pilot Studies on Effects of Musical Agency Intervention on Cognitive Functioning in Participants with Dementia and Stroke Survivors

In a recent pilot study with thirty-eight residents of local urban long-term care facilities (30 female, 8 male; *n* = 27 complete datasets used in statistical analysis), the effects of the active music intervention Jymmin on cognitive and emotional parameters were investigated and reported in a master’s thesis [35]. Participants were older adults (mean age = 81.97, SD = 7.04, range 63–92) with different stages of dementia, as classified based on cognitive screening scores Mini-Mental State Examination, (MMSE) [36]. Thirteen participants were classified as having mild dementia (MMSE = >18), sixteen participants as having moderate dementia (MMSE = 10–17), and eight as having severe dementia (MMSE < 10). By using a cross-over design, all participants were randomly assigned to receive six minutes of either the musical agency intervention (using rubber bands) or the control (rubber band exercise with passive music listening) across the first five consecutive days (Monday to Friday) and then performed the other condition for another five consecutive days (Monday to Friday). Both conditions were performed in groups of two participants and the baseline measures were assessed prior to the training. Outcome measures were cognitive capabilities across different domains, including selective attention, processing speed, memory (short- and long-term delay), confrontation naming, and motor coordination (assessed using The Short Test Capturing Cognitive Performances [37]; three versions for repeated measures (A, B, and C)). Additionally, the effects on mood were assessed by using the Multidimensional Mood Questionnaire (MDMQ; Mehrdimensionaler Befindlichkeitsfragebogen [38]) consisting of three subscales including “Good-Bad Mood”, “Alertness-Tiredness”, and “Calmness-Agitation”. Cognitive capabilities and current mood measures were first obtained at baseline, second after the first five days of intervention (on the respective sixth day when there was no intervention), and third after the following five days of having performed the respective other intervention (again on the respective sixth day when there was no intervention). The results showed significant improvements in scores on a confrontation naming task across both conditions and significant improvements in a short-term memory task for the musical agency condition. No significant changes were observed in mood ratings on the day consecutive to the intervention. The findings of this study underline that active music interventions seem to stimulate cognitive functioning stronger than passive music listening in participants with dementia. Most interestingly, this effect is observed even if physical engagement is held constant for the active and passive music intervention. Therefore, musical agency seems to be a key factor in driving the beneficial effects on cognitive functioning.

Similar results were reported in another pilot study in a master’s thesis, which investigated the short-term effects of a musical agency intervention on cognitive capabilities in participants who have suffered from stroke [39]. In total, thirteen participants (mean age = 58.38, SD = 8.96, range = 43–70, of which, eight complete datasets could be included in the statistical analysis) were included in the study, who suffered any kind of stroke in any part of the brain (for a full description of stroke etiologies, territory, and hemisphere, see the original master’s thesis). Further inclusion criteria were the time since injury (3–24 months) and age (18–75 years). Exclusion criteria were as follows: severe mobility issues, dementia, and aphasia. Note, that the mean time since the stroke in participants was 60 weeks (SD = 21.14, range = 36–95 weeks), which suggests that deficits in these stroke survivors have to be regarded as chronic. All participants performed the musical agency condition Jymmin and a control condition (music listening while being physically engaged) using Thera-Band^®^ rubber bands in a cross-over design (participants were randomly assigned to an order of conditions). Each condition lasted ten minutes. The music presented during the control condition was the same music that was recorded from one of the groups during the Jymmin performance. Note that different levels of difficulty were available so that the intervention could be tailored to individual needs. Cognitive capabilities and other measures were first obtained at baseline, second after the first ten minutes of having performed one condition, and third after the following ten minutes of having performed the respective other condition. Participation in both conditions took place on the same day. The cognitive measures included the following: working memory (assessed using the digit span backward (DSB) subtest of the Wechsler Memory Scale—Revised [40] inhibitory control [41], and divergent thinking (as measured by Guilford Alternative Uses Task [34]). Furthermore, the feasibility of the Jymmin intervention was assessed by using subjective ratings on different aspects of the invention with 5-point Likert scales. The results showed a significant improvement in working memory capacity after the musical agency condition (Jymmin). This is especially interesting as existing research on the effects of music interventions on working memory is rather scarce. While a few studies indicate positive tendencies for improved working memory after music interventions (e.g., Särkämö et al., 2008 [42]), the research to date is not extensive enough to draw reliable and robust conclusions [43]. The results of this study can therefore contribute to a better understanding of working memory effects due to active music interventions. Inhibitory control and divergent thinking capabilities did not differ significantly between both conditions. In subjective ratings concerning the feasibility of the study, the participants liked the musical agency intervention overall (M = 4.13, SD = 0.84) and all participants indicated that the duration of the intervention was doable. Six participants (75%) indicated that they could imagine doing the Jymmin intervention on a regular basis (at different frequencies). Of the participants included in the data analysis, six (75%) wanted to be contacted in the future for further studies using the Jymmin intervention.

### 4.2. Study Addressing the Influence of Active Music Intervention on Emotional Parameters in Patients with Chronic Pain

As previous research showed that active music interventions can have a positive effect on mood. A study as part of a master’s thesis investigated whether active engagement in music-making is more beneficial than music listening in terms of anxiety and pain levels during physical activity [44], which is often avoided in patients with chronic pain [45].The experience of anxiety is especially known to be central to the development of chronic pain [46] and music listening was previously shown to exert analgesic effects [47]. Again, we applied the previously described music feedback paradigm and explored this method as an intervention to potentially reduce anxiety in a group of patients with chronic pain (N = 24, 20 female and 4 men; age range 34–64, M = 51.67, SD = 6.84) and with various anxiety levels (assessed using the State-Trait Anxiety Inventory [48].All participants underwent an extensive baseline assessment of pain characteristics, co-morbidities, and relevant psychological parameters (anxiety, mood, self-efficacy). Then, all participants performed two conditions (the order of conditions was balanced and randomized): one condition, namely, Jymmin, where exercise equipment was modified with music feedback so that it could be played like musical instruments by groups of three; and second, a conventional workout condition where groups of three performed exercise on the same devices but where they listened to the same type of music passively. Participants’ levels of anxiety, mood, pain, and self-efficacy were assessed with standardized psychological questionnaires before the experiment and after each condition. Results demonstrate that exercise with musical feedback reduced anxiety values in patients with chronic pain significantly as compared to a conventional workout with passive music listening with a medium effect size (r = 0.43). There were no significant overall changes in pain, but patients with greater anxiety levels compared to those with moderate anxiety levels were observed to potentially benefit more from the music feedback intervention in terms of the alleviation of pain. Mood and self-efficacy did not differ significantly between conditions. Furthermore, it was observed that patients during Jymmin treatment more strongly perceived motivation through others with a medium effect size (r = 0.41).

## 5. Conclusions

In conclusion, in this paper, we reviewed studies that employed active music therapy in patient populations. We noted that based on the above and similar observations, music therapy is recommended as a non-medicated therapy. For example, the American Heart Association and the American Stroke Association classify music intervention in the category “Class IIb, Level B”, which indicates that it is recommended, although its efficacy needs to be further established [49] illustrating the key problem in this field. This is the lack of replicability in terms of various aspects of music therapy, e.g., degree of physical exertion, musical agency, or systematic matching between control and patient groups. We presented an active music intervention paradigm (i.e., Jymmin) that has the potential to address these caveats. We outlined several parameters that either can be varied or need to be controlled for when investigating the effects of music-making in therapy using this paradigm. Further, we reviewed a series of experiments employing the Jymmin paradigm that allows for precisely taking into account the amount of physical activity exerted by participants and creating control conditions that are comparable with respect to the level of activity. Our findings outline several benefits of active musical agency in rehabilitation therapy. These include improved mood or increased motivation to engage in strenuous procedures during rehabilitation. The capacity to actively control music during the interventions led to an increase in mood; divergent thinking capacity; muscle efficiency; and reductions in anxiety, pain, and perceived effort. We found that active music interventions should be more broadly integrated into therapeutic plans, but that including control conditions that are comparable with respect to activity level is key to an understanding of underlying mechanisms.

## Data Availability

Not applicable.
